# Development of a hypoxia-responsive macrophage prognostic model using single-cell and bulk RNA sequencing in pancreatic cancer

**DOI:** 10.1371/journal.pone.0322618

**Published:** 2025-05-02

**Authors:** Heming Ge, Gerrit Wolters-Eisfeld, Thilo Hackert, Yuqiang Li, Cenap Güngör

**Affiliations:** 1 Department of General, Visceral and Thoracic Surgery, University Medical Center Hamburg-Eppendorf, Hamburg, Germany; 2 Department of General Surgery, Xiangya Hospital, Central South University, Changsha, China; University of Nebraska Medical Center, UNITED STATES OF AMERICA

## Abstract

**Objective:**

Pancreatic ductal adenocarcinoma (PDAC) is characterized by a low survival rate and limited responsiveness to current therapies. The role of hypoxia in the tumor microenvironment is critical, influencing tumor progression and therapy resistance. The aim of this study was to implement the complex dynamics of the hypoxic tumor microenvironment in PDAC in a hypoxia-related prognosis model.

**Methods:**

We utilized single-cell RNA sequencing (scRNA-seq) data and integrated it with TCGA-PAAD database to identify hypoxia-responsive macrophage subsets and related genes. Kaplan-Meier survival analysis, Cox regression, and Lasso regression methods were employed to construct and validate a hypoxia-related prognostic model. The model’s effectiveness was evaluated through its predictive capabilities regarding chemotherapy sensitivity and overall survival.

**Results:**

Our research integrated data from scRNA-seq and the TCGA-PAAD database to construct a hypoxia-related prognostic model that encompassed 13 critical genes. This hypoxia model independently predicted chemotherapy response and poor outcomes, outperforming traditional clinicopathologic features. Additionally, a pan-cancer analysis affirmed the relevance of our hypoxia-related genes across multiple malignancies, particularly highlighting *KRTCAP2* as a pivotal biomarker associated with worse prognosis and reduced immune infiltration.

**Conclusion:**

Our findings underscored the prognostic potential of hypoxia-related model and offered a novel avenue for therapeutic targeting, aiming to ameliorate outcomes in pancreatic cancer.

## Introduction

Pancreatic cancer is globally recognized as one of the most lethal malignancies [1], with ductal adenocarcinoma (PDAC) representing the predominant pathological type [2]. Despite noteworthy advancements in the field of PDAC research, the five-year survival rate for patients still remains approximately 10% [1]. Current systemic therapies encompassing surgical intervention, chemotherapy, and immunotherapy are widely administered; however, the response rate among patients is still extremely low [3,4]. Notably, even though immune checkpoint inhibitors have markedly enhanced outcomes in a variety of tumor types recently, their efficacy in improving pancreatic cancer prognosis remains limited [5]. Consequently, there is a pressing need to identify novel molecular markers and develop effective therapeutic targets for pancreatic cancer.

Hypoxia is a hallmark of the tumor microenvironment in solid malignancies, including pancreatic cancer, where it contributes to several pathological processes such as impaired immune responses, metabolic reprogramming, epithelial-mesenchymal transition, and increased therapy resistance [6,7]. In pancreatic cancer, the presence of hypoxic areas is critically implicated in adverse patient outcomes and serve as independent prognostic markers [8]. Moreover, tumor-associated macrophages (TAMs) are notably accumulated in these hypoxic regions of the tumor. Previous studies have demonstrated that TAMs not only facilitate tumorigenesis but also correlate with poor survival outcomes due to their involvement in inflammation, further emphasizing their potential as therapeutic targets in pancreatic cancer [9,10]. The dynamic interactions between cancer cells and TAMs within these hypoxic environments are pivotal in driving tumorigenesis and therefore present promising targets for innovative therapeutic strategies in cancer management [11].

In this study, our findings indicated that hypoxia significantly affects macrophages more than any other investigated immune cell type within the PDAC microenvironment. We pinpointed a distinct subset of macrophages using single-cell RNA sequencing (scRNA-seq) data from pancreatic cancer, which displayed heightened susceptibility to hypoxic conditions. By integrating data from the TCGA-PAAD database, we identified a subset of hypoxia-related genes and subsequently developed a novel hypoxia-related prognostic model. This model is independent from current clinicopathologic features in pancreatic cancer. We further validated the prognostic efficacy of our model across multiple public databases. Our results confirmed that this model effectively predicts the sensitivity to common chemotherapeutic agents, including gemcitabine, oxaliplatin, cisplatin, 5-fluorouracil, and paclitaxel, within the pancreatic cancer context. Additionally, the model demonstrated robust prognostic prediction capabilities in a pan-cancer analysis.

## Materials and methods

### Data acquisition

The 200 hypoxia hallmark genes were retrieved from the Molecular Signatures Database (https://www.gsea-msigdb.org/gsea/msigdb/index.jsp). According to the MSigDB, this set consists of genes that are up-regulated in response to low oxygen levels (hypoxia).

The scRNA-seq data GSE155698 [12] were obtained from the Gene Expression Omnibus (GEO) database (https://www.ncbi.nlm.nih.gov/geo/), which comprised 19 samples: 16 primary PDAC tissues and 3 non-malignant pancreas tissues.

Bulk RNA-seq data, along with copy number variation (CNV), single-nucleotide variants (SNV), and associated clinicopathologic information of PAAD were accessed from the Cancer Genome Atlas (TCGA) database (https://portal.gdc.cancer.gov/), which included 159 pancreatic cancer tissues, following the exclusion of samples lacking survival data and clinical details. For external validation, databases from two cohorts, PACA-CA and PACA-AU, were utilized, encompassing 142 and 76 samples respectively, after removing samples without follow-up. These were accessed from the International Cancer Genome Consortium (ICGC) database (https://dcc.icgc.org/).

### Processing of scRNA-seq data

ScRNA-seq data were processed using the R package “Seurat” (v5.0.1). Initial quality control was performed with the following criteria:

1) Genes expressed in fewer than three single cells were excluded;
2) Cells with total gene counts <200 or > 5,000 were removed to filter low-quality cells and potential doublets;
3) Cells with mitochondrial gene content >20% were discarded to eliminate apoptotic cells.

#### Normalization and feature selection.

Gene expression was normalized using the LogNormalize method (scale factor = 10,000) via the NormalizeData function. To identify highly variable genes for downstream analysis, 2,000 variable features were selected using the FindVariableFeatures function with the “vst” method.

#### Data scaling and dimensionality reduction.

Expression values were standardized (z-score transformation) using ScaleData. Principal component analysis (PCA) was performed on scaled data (RunPCA), and the top 30 principal components (PCs) were selected based on the elbow plot for downstream clustering.

#### Cell clustering and annotation.

Cell neighborhoods were constructed using FindNeighbors (dims = 1:30), followed by FindClusters (resolution = 0.4) to partition cells into distinct clusters. Uniform Manifold Approximation and Projection (UMAP) was applied for 2D visualization (RunUMAP, dims = 1:30). Cluster identity was determined by manually curating the top 10 marker genes from FindAllMarkers (Wilcoxon test, min.pct = 0.25, logfc.threshold = 0.25) against canonical cell-type markers (Fig 2D).

**Fig 1:**
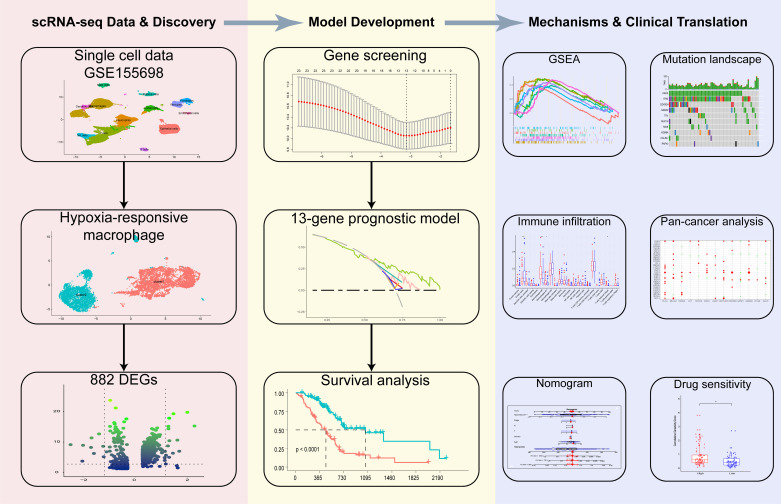
Flow chart of this study.

### Calculation of hypoxic microenvironment scores

#### For bulk RNA-seq data (TCGA-PAAD).

The tumor hypoxic microenvironment scores were computed via single-sample Gene Set Enrichment Analysis (ssGSEA) using the GSVA R package (v1.46.0). The 200 hypoxia hallmark genes were ranked per sample, and enrichment scores were calculated with 1,000 permutations. All 200 genes were included in the analysis. Subsequently, samples were classified into high and low hypoxic microenvironment groups based on the median score.

#### For scRNA-seq data.

Hypoxia activity was quantified using two methods

##### AddModuleScore:

The average expression of hypoxia genes was subtracted by the mean expression of 100 control gene sets to generate per-cell hypoxia scores.

##### AUCell:

Gene rankings per cell were computed using the AUCell R package (v 1.20.2), and area under the curve (AUC) values were computed using the top 10% of ranked genes.

### Gene set enrichment analysis

To investigate differential signaling pathways, we performed Gene set enrichment analysis (GSEA) using the package “clusterProfiler” (v4.6.0). This analysis compared macrophage cluster 1 with macrophage cluster 2 in scRNA-seq data, as well as high and low hypoxia groups within the TCGA-PAAD dataset. Pathway marker genes were downloaded from the MSigDB. The analysis was performed with the permutations parameter set to 1000. Results were considered significant if the normalized enrichment score (NES) had an absolute value greater than 1, with a FDR below 0.25 and p-values less than 0.05.

### Construction and evaluation of the prognostic model

The FindMarkers function within the package “Seurat” was employed to identify DEGs between normal and tumor tissues in macrophage cluster 1 in scRNA-seq data, using Wilcoxon tests with a threshold value of 0.25. Genes were further filtered based on statistical significance and effect size, retaining only those with a p-value less than 0.05 and an absolute log2 fold change (|log2FC|) greater than 0.25. A univariate Cox regression analysis was performed to evaluate the prognostic value of these DEGs for OS in patients from the TCGA-PAAD dataset using the package “survival” (v3.4.0) [13]. Subsequent least absolute shrinkage and selection operator (LASSO) regression with 10-fold cross-validation (glmnet, v4.1.8) was used to penalize overfitting genes [14–16]. The optimal lambda (λ = 0.0432) was selected via minimum partial likelihood deviance.

Utilizing the 13 hypoxia-related genes, ssGSEA was performed to calculate the enrichment fraction of hypoxia in each sample from the TCGA-PAAD database. Patients were stratified into high and low hypoxia groups based on the median hypoxia score. The prognostic efficacy of the hypoxia model was validated through the construction of receiver operating characteristic (ROC) curves and Kaplan–Meier (K–M) survival curves, using the packages “timeROC” (v0.4) and “survminer” (v0.4.9), respectively. Decision curve analysis (ggDCA, v1.2) was used to assess clinical utility. Additionally, the validity of the model was corroborated using external databases from PACA-CA and PACA-AU.

### Mutation landscape analysis

SNV and TMB analysis: Mutation annotation format files were processed using package “maftools” (v2.14.0). Tumor mutational burden (TMB) was calculated as mutations per megabase. CNV analysis: Segmented copy number data from TCGA were analyzed using GISTIC 2.0 (thresholds: amplification = 0.2, deletion = -0.2).

### Immune landscape analysis

To investigate the characteristics of immune cells across different hypoxia groups, we employed the R package “estimate” (v1.0.13) to calculate immune and stromal scores. Furthermore, we adopted the CIBERSORT algorithm [17] to evaluate the infiltration of 22 immune cell types (LM22 signature matrix) in both high and low hypoxia groups using package “CIBERSORT” (v0.1.0). The association between the hypoxia score and the levels of immune cell infiltration was quantified using the Pearson correlation coefficient.

### Drug sensitivity analysis

IC50 values for drugs were determined from the Genomics of Drug Sensitivity in Cancer (GDSC) database using the R package “oncoPredict” (v0.2). Differences in IC50 values between high and low hypoxia groups were statistically analyzed using the Wilcoxon test to assess significance.

### Statistical analysis

All statistical analysis were performed using R software (version 4.2.2). Differences among groups were evaluated using the Wilcoxon test, while correlations were assessed using either the Spearman or Pearson correlation coefficients. Survival differences were analyzed using the Log-rank test through Kaplan-Meier curves. All statistical tests were two-tailed, and a P-value of less than 0.05 was considered statistically significant.

## Results

### Association between tumor hypoxic microenvironment and survival outcomes in pancreatic cancer

**Fig 2:**
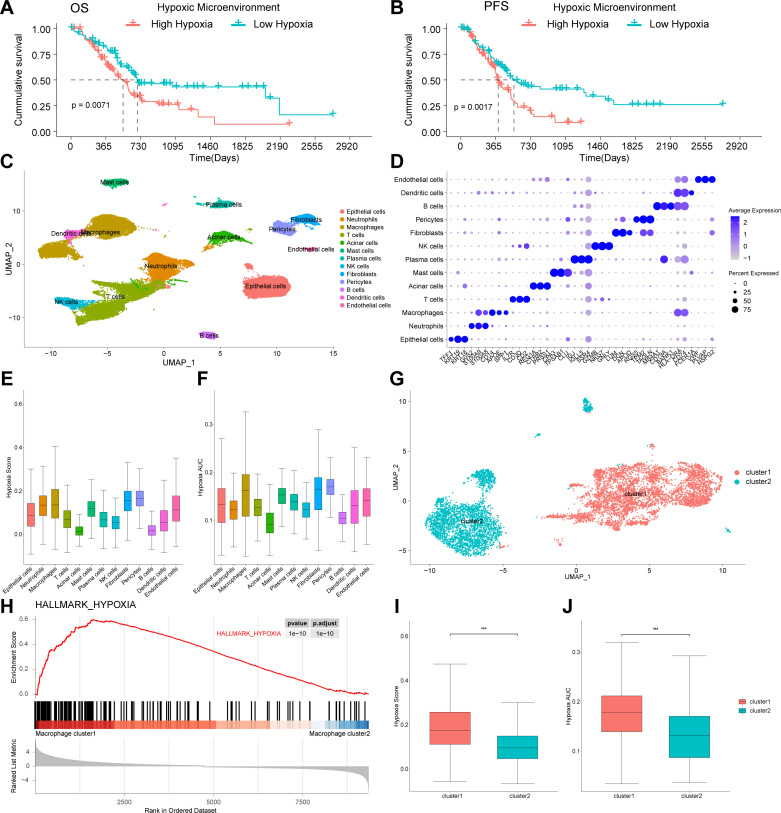
Single-cell RNA-sequencing analysis identified hypoxia-responsive macrophage subcluster. **(A, B)** Prognostic differences of the tumor hypoxic microenvironment in TCGA-PAAD cohort. **(C)** UMAP plot of 13 cell types in PDAC samples. **(D)** Key cell markers used to identify cell types. **(E, F)** Hypoxic microenvironment scores calculated by the AddModuleScore and AUCell function. **(G)** UMAP plot of macrophage subclusters. **(H)** GSEA showed that Hypoxia pathways was activated in macrophage cluster 1. **(I, J)** Hypoxic microenvironment scores calculated by the AddModuleScore and AUCell function in macrophage subclusters. Data sources: Panels A and B use the TCGA-PAAD cohort. Panels C-J use the dataset GSE155698.

The 200 hypoxia hallmark genes were retrieved from the Molecular Signatures Database [18]. We performed Kaplan−Meier survival analysis to explore the correlation between the tumor hypoxic microenvironment and survival outcomes in TCGA-PAAD. Our findings demonstrated that a highly tumor hypoxic microenvironment in pancreatic cancer correlated with adverse prognostic outcomes, including reduced overall survival (OS) and progression free survival (PFS) (Figs 1, 2A and 2B).

### Annotation of cell types and hypoxic microenvironment scores

Based on scRNA-seq data from GSE155698 [12], we obtained gene expression profiles of 37,018 cells from 16 primary PDAC samples and 7,316 cells from 3 non-malignant pancreas samples for further analysis after rigorous quality control and batch correction. Cells were classified into 13 distinct types based on the expression of typical cell markers. These types included epithelial cells, neutrophils, macrophages, T cells, acinar cells, mast cells, plasma cells, NK cells, fibroblasts, pericytes, B cells, dendritic cells, and endothelial cells (Fig 2C). Key marker genes utilized for annotation are depicted in Fig 2D.

We subsequently calculated hypoxic microenvironment scores for all identified cell types using the AddmoduleScore and AUCell functions. The analysis revealed that macrophages exhibited the highest hypoxic microenvironment scores, significantly higher than those of other immune cell types (Fig 2E and 2F). This significant elevation among macrophages suggested that tumor hypoxic microenvironment may play a crucial role in modulating macrophage functions, thereby potentially impacting the progression of PDAC. To further dissect the sensitivity of macrophages to the tumor hypoxic microenvironment, we performed subclustering of macrophages and recalculated their hypoxic microenvironment scores. We identified a subgroup of macrophages displaying significantly elevated hypoxic microenvironment scores, which we termed “macrophage cluster1”, representing hypoxia-responsive macrophages. In contrast, other subgroups exhibited relatively lower sensitivity to tumor hypoxic microenvironment, and we designated these as “macrophage cluster2” (Fig 2G). Gene Set Enrichment Analysis (GSEA) conducted on these subtypes indicated a significantly more pronounced effect of hypoxia on macrophage cluster1, compared to cluster2 (Fig 2H), consistent with the hypoxic microenvironment scores calculated by AddmoduleScore (Fig 2I) and AUCell functions (Fig 2J).

### Hub genes identification and hypoxia signature construction

**Fig 3:**
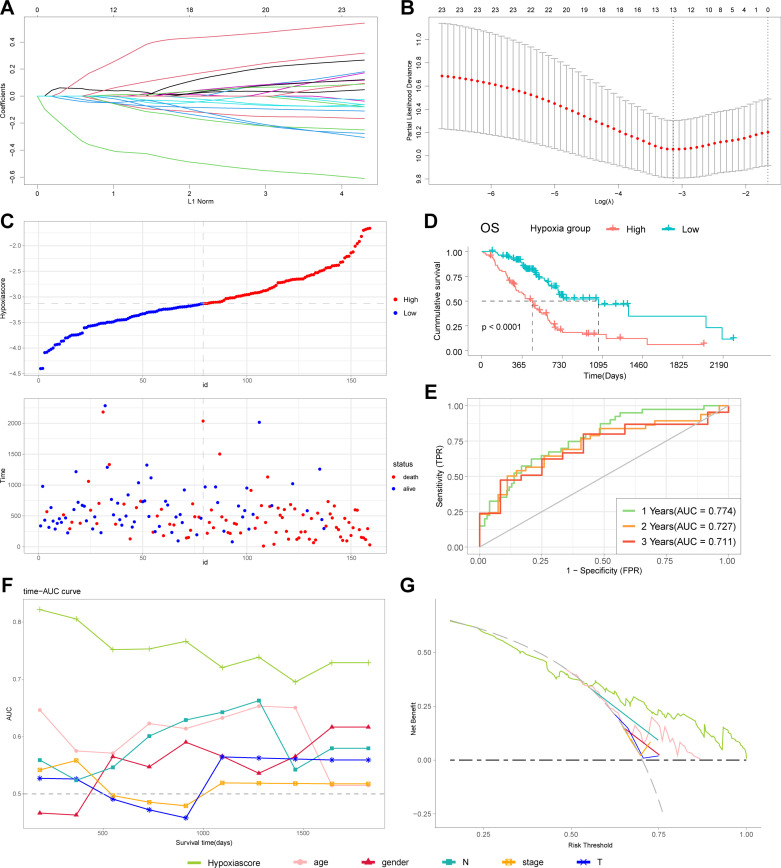
Construction of hypoxia-related prognostic model in TCGA cohort. **(A, B)** LASSO regression of hypoxia-related genes. **(C)** Relationship between survival status and hypoxia scores in TCGA cohort. **(D)** Kaplan−Meier curves of patients in high and low hypoxia groups. **(E)** ROC curves of hypoxia model for predicting the risk of death at 1, 2, and 3 years. **(F)** Time-AUC curves evaluating the predictive capacity of the hypoxia model and clinicopathologic features. **(G)** Decision curve analysis evaluating the benefit rate of patients receiving clinical treatment based on the hypoxia model and clinicopathologic features. Data sources: Panels A-G use the TCGA-PAAD cohort.

To develop a risk signature, we initially identified differentially expressed genes (DEGs) between PDAC and non-malignant pancreas tissues within macrophage cluster1 in scRNA-seq data. A total of 882 DEGs were discerned, comprising 571 upregulated and 311 downregulated genes. These DEGs represented the specific alterations in hypoxia-responsive macrophages within pancreatic cancer. Subsequently, we evaluated the prognostic value of these 882 DEGs using univariate Cox regression analysis, identifying 23 genes associated with poor prognosis in the TCGA-PAAD cohort. Further refinement was conducted through Lasso Cox regression analysis to pinpoint hub genes. As illustrated in Fig 3A and 3B, the optimal Lambda is 0.0432, and 13 hypoxia-related genes were finally included in the construction of the hypoxia-related prognostic model, including *LYZ*, *SCN1B*, *PLAU*, *INSIG2*, *DSC2*, *MICAL1*, *U2AF1*, *KRTCAP2*, *DDX60L*, *SATB1*, *SAMD9*, *LTC4S*, *IGLL5*. The regression coefficients for each gene were detailed in S1 Table.

### Hypoxia model predicts survival in TCGA cohort

To assess the prognostic significance of hypoxia in PDAC, we calculated a hypoxia score for each patient in the TCGA-PAAD cohort according to the hypoxia-related prognostic model. The hypoxia score represented the degree of hypoxic activity based on the hypoxia-related prognostic model. Patients were stratified into high and low hypoxia groups using the median hypoxia score as a cutoff. Fig 3C displayed the distribution of hypoxia scores and correlated them with patient survival status, indicating a higher mortality rate in the high hypoxia group. Kaplan−Meier survival curve demonstrated that patients with high hypoxia scores exhibited significantly worse outcomes, compared to those with low scores (Fig 3D). Additionally, to assess the predictive efficacy of our hypoxia model, time-dependent ROC curves for OS were generated. The area under the curve (AUC) values were 0.774 at 1 year, 0.727 at 2 years, and 0.711 at 3 years (Fig 3E). These values were significantly superior to those derived from clinicopathologic characteristics alone (Fig 3F), underscoring the model’s excellent predictive capability. Decision curve analysis confirmed these findings, demonstrating that clinical interventions guided by the hypoxia model yielded greater benefits compared to those based solely on clinicopathologic characteristics (Fig 3G). Similar results were also observed in external validation cohorts, including PACA-CA (S1 Fig) and PACA-AU (S2 Fig).

### Analysis of clinicopathologic characteristics and nomogram construction

**Fig 4:**
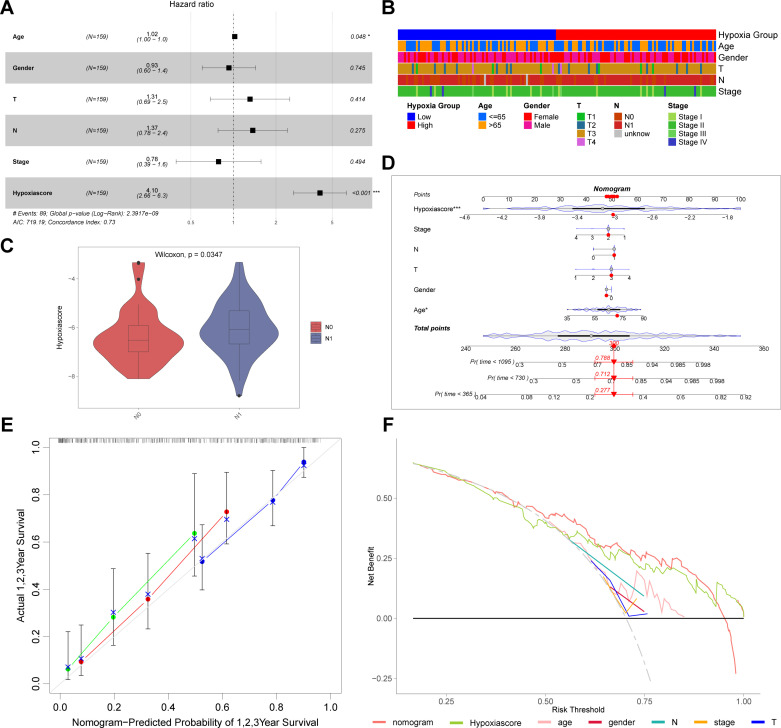
Clinicopathologic features of hypoxia model and construction of prognostic nomogram. **(A)** Multivariate Cox regression analysis of hypoxia model and clinicopathologic characteristics. **(B)** Heatmap of hypoxia groups and clinicopathologic characteristics. **(C)** Relationship between hypoxia score and N stage of patients. **(D)** Construction of the nomogram integrating the hypoxia model and clinicopathologic characteristics. **(E)** Calibration curves for 1, 2, and 3 years of nomogram. **(F)** Decision curve analysis evaluating the benefit rate of patients receiving clinical treatment based on the nomogram. Data sources: Panels A-F use the TCGA-PAAD cohort.

To ascertain whether the hypoxia score could independently influence prognosis, we collected clinicopathologic data from the TCGA-PAAD cohort. Multivariate Cox regression analysis confirmed that the hypoxia score was an independent prognostic factor for patients with pancreatic cancer (Fig 4A and S3 Fig). We compared clinicopathologic characteristics between the high and low hypoxia groups; a heatmap analysis indicated comparable features across both groups (Fig 4B). This suggested that the prognostic capability of the hypoxia model operated independently of clinicopathologic characteristics. Further analysis assessed the differences in hypoxia scores across various clinicopathologic factors; notably, higher N stages were associated with increased hypoxia scores (Fig 4C and S3 Fig). Additionally, stratified analysis based on clinicopathologic characteristics such as age<=65, age > 65, Male, T3-T4, N0, N1, Stage Ⅰ-Ⅱ, Stage Ⅲ-Ⅳ demonstrated significant differences in survival outcomes between the high and low hypoxia groups (S4 Fig).

Finally, we developed a novel nomogram that integrated clinicopathologic features and hypoxia scores (Fig 4D). This nomogram exhibited strong predictive power for survival outcomes, as evidenced by the calibration curve (Fig 4E). Additionally, we conducted decision curve analysis to evaluate the nomogram’s predictive efficacy, and the results demonstrated that the benefit rate for patients treated based on the nomogram exceeded those who were treated based on other clinicopathologic features alone (Fig 4F).

### Gene set enrichment analysis

**Fig 5:**
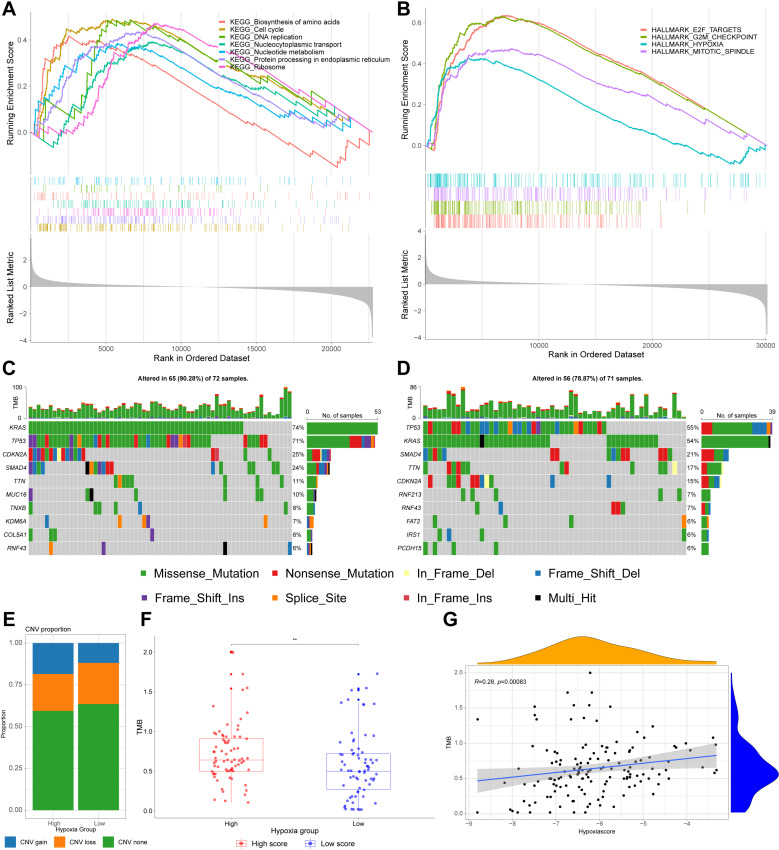
Molecular features of high and low hypoxia groups. **(A, B)** GSEA analysis revealed enriched signaling pathways in high hypoxia group. **(C, D)** Waterfall diagrams displaying SNV mutations of high and low hypoxia groups. **(E)** Differential proportion of CNV mutations in high and low hypoxia groups. **(F)** The comparison of TMB between high and low hypoxia groups. **(G)** The Pearson correlation analysis of TMB and hypoxia scores. Data sources: Panels A-G use the TCGA-PAAD cohort.

To elucidate the molecular mechanisms underlying the differences in cancer progression between high and low hypoxia groups, we performed Gene Set Enrichment Analysis (GSEA). The analysis revealed that the high hypoxia group exhibited significant enrichment in several critical pathways, including biosynthesis of amino acids, cell cycle, DNA replication, nucleocytoplasmic transport, nucleotide metabolism, protein processing in the endoplasmic reticulum, ribosome, E2F targets, G2M checkpoint, hypoxia and mitotic spindle (Fig 5A and 5B).

### Mutation landscape analysis

Gene mutations play a critical role in tumor development and resistance to therapy. Analysis of single-nucleotide variants (SNV) indicated that missense mutations were the most common type in both the high and low hypoxia groups. Despite the top five most frequently mutated genes - *KRAS*, *TP53*, *CDKN2A*, *SMAD4* and *TTN* - being identical between the groups, the overall mutation rate was significantly higher in the high hypoxia group (90.28%), compared to the low hypoxia group (78.87%), as shown in Fig 5C and 5D. Further investigations revealed a higher incidence of copy number variation (CNV) gains in the high hypoxia group (Fig 5E). Additionally, the high hypoxia group harbored a remarkably greater tumor mutational burden (TMB) than the low hypoxia group (Fig 5F), and the hypoxia score was positively correlated with TMB, with a correlation coefficient of 0.28 and a statistically significant level (P < 0.001), as illustrated in Fig 5G. It is widely accepted that TMB is associated with poor prognosis, which may be the reason for the poor prognosis in the high hypoxia group.

### Unveiling immune cell infiltration in hypoxia model

**Fig 6:**
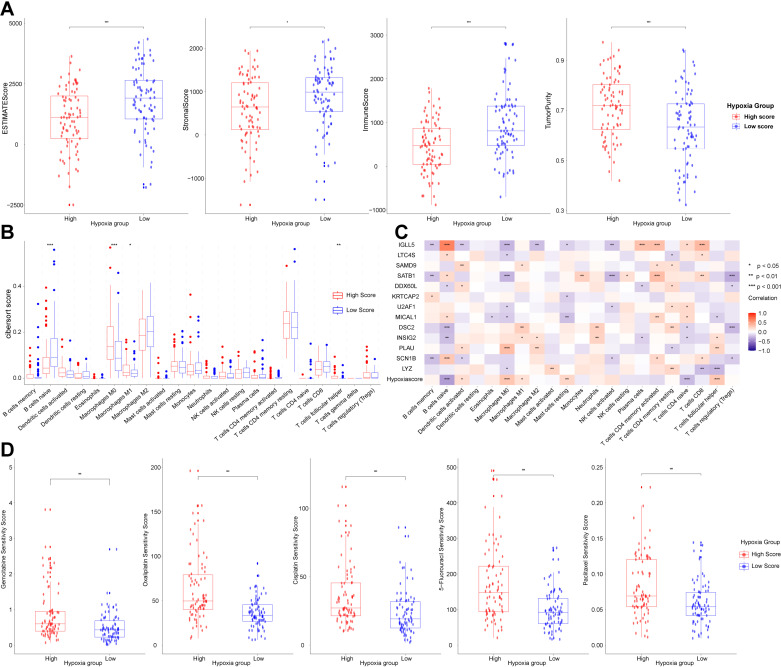
Relationship of hypoxia score with immune cell infiltration and chemotherapy sensitivity. **(A)** Differences among immune score, stromal score, ESTIMATE score, and tumor purity between different hypoxia groups. **(B)** Differential proportion of 22 immune cell subtypes between high and low hypoxia groups by the CIBERSORT algorithm. **(C)** Correlations between hypoxia-related genes and 22 immune cell subtypes. **(D)** Correlation analysis between hypoxia score and chemotherapy sensitivity. Data sources: Panels A-C use the TCGA-PAAD cohort. Panel D uses TCGA-PAAD cohort and GDSC dataset.

The immune microenvironment plays a crucial role in the prognosis of pancreatic cancer patients. We explored the association between hypoxia scores and immune cell infiltration using the ESTIMATE algorithm, which assesses the level of immune cell presence within tumors. Our analysis indicated that the high hypoxia group exhibited higher tumor purity and lower immune, stromal, and ESTIMATE scores (Fig 6A), suggesting a negative correlation between hypoxia scores and immune cell infiltration. Moreover, the CIBERSORT algorithm was employed to further analyze the composition of immune cells in the TCGA-PAAD cohort. The findings demonstrated a reduced presence of anti-tumor immune cells such as naive B cells in the high hypoxia group, while macrophages M0 were predominantly enriched (Fig 6B), indicating that the hypoxia score was closely associated with an immunosuppressive tumor microenvironment. Correlation analysis between hypoxia-related genes and immune cell infiltration levels further substantiated these observations (Fig 6C).

### Drug sensitivity analysis

We further explored the correlation between hypoxia score and chemotherapy sensitivity in pancreatic cancer. Utilizing the GDSC database, we assessed the response to chemotherapeutic agents. Notably, most drugs exhibited significant differential responses between the high and low hypoxia groups, including key agents such as gemcitabine, oxaliplatin, cisplatin, 5-Fluorouracil and paclitaxel (Fig 6D). The elevated half-maximal inhibitory concentrations (IC50) of these drugs in the high hypoxia group suggested a diminished chemotherapy efficacy, which may contribute to the observed poorer prognosis in this group.

### Pan-cancer analysis

**Fig 7:**
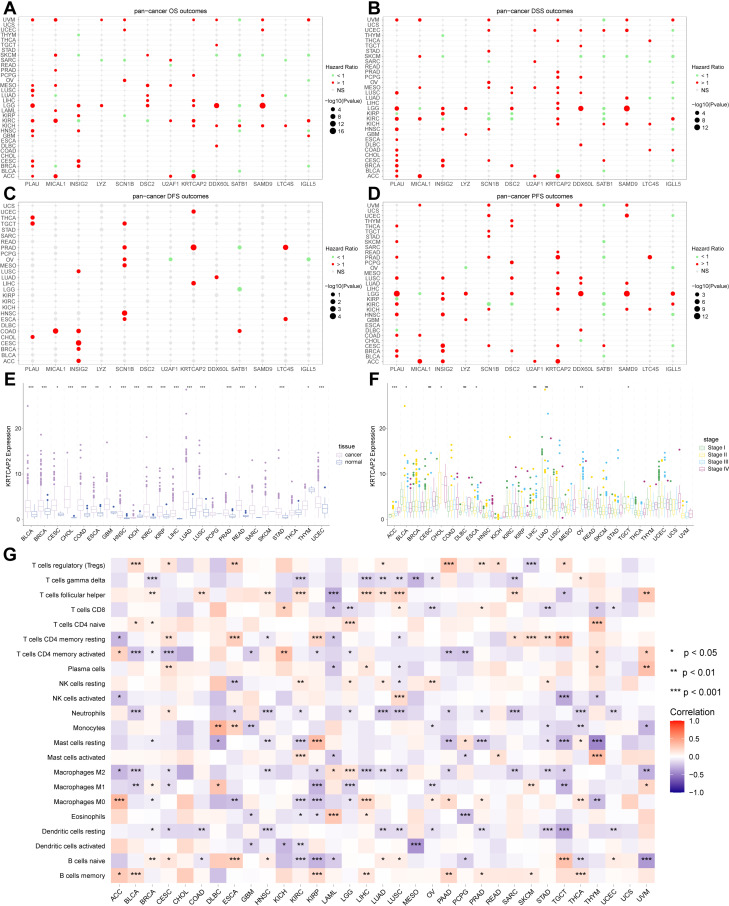
Pan-cancer analysis of hypoxia-related genes. **(A-D)** Relationship between hypoxia-related genes with OS **(A)**, DSS **(B)**, DFS (C) and PFS (D) across multiple cancer types. **(E)** Differences in *KRTCAP2* expression levels between cancer and adjacent normal tissues across various tumor types. **(F)** Differences in *KRTCAP2* expression levels between different tumor stages across various tumor types. **(G)** The relationship between *KRTCAP2* and immune cell infiltration across various tumor types. Data sources: Panels A-G use the TCGA dataset.

We extended our analysis of hypoxia-related genes to a pan-cancer analysis. These genes were found to be significantly associated with various survival metrics across multiple cancer types, including overall survival (OS) (Fig 7A), disease specific survival (DSS) (Fig 7B), disease free survival (DFS) (Fig 7C), and progression free survival (PFS) (Fig 7D). Among these, *KRTCAP2* consistently emerged as a prognostic marker, with its expression linked to unfavorable outcomes across nearly all investigated cancer types, evidenced by a hazard ratio (HR) greater than 1. We further examined *KRTCAP2* expression levels in cancer tissues, compared to adjacent normal tissues, and across different stages of cancer progression. The results indicated a significant overexpression of *KRTCAP2* in cancer tissues across most cancer types (Fig 7E), with its abundant expression escalating in higher cancer stages (Fig 7F). Additionally, we also assessed the correlation between *KRTCAP2* expression and immune cell infiltration across various tumors and found *KRTCAP2* expression exhibited a negative correlation with several immune cell populations, including T cells gamma delta, CD8^+^ T cells, CD4^+^ memory activated T cells, neutrophils, monocytes, resting mast cells, M1 macrophages, and activated dendritic cells, across multiple tumor types. Conversely, a positive correlation was observed between *KRTCAP2* expression and the immunosuppressive Treg cells (Fig 7G), suggesting that *KRTCAP2* played a significant role in modulating the tumor immune microenvironment across diverse cancer types.

## Discussion

The incidence of pancreatic cancer has been steadily increasing, with projections indicating that this upward trend will continue for the foreseeable future in both the United States and Europe [19]. Rahib *et al.* have projected that PDAC will become the second-leading cause of cancer-related mortality in the United States by 2030, despite rapid advancements in cancer therapeutics [20]. Therefore, identifying the factors contributing to pancreatic cancer drug resistance is imperative. Current literature highlights that pancreatic cancer is notably hypoxic compared to other cancer types, with the hypoxic microenvironment significantly enhancing drug resistance [21–23]. Despite these insights, current research has not yet revealed the specific cell types within the pancreatic cancer microenvironment that are most likely influenced by the local hypoxic microenvironment.

In this study, we observed that macrophages are disproportionately affected by hypoxic conditions compared to other immune cell types. Macrophages are indispensable components of the tumor microenvironment and play pivotal roles in the progression, metastasis, and therapeutic resistance of PDAC, thus posing significant challenges to developing effective treatment strategies targeting this deadly cancer [24,25]. Furthermore, the hypoxic microenvironment is known to promote macrophage polarization and their enrichment within tumors [26]. Our subgroup analysis further identified macrophage cluster1 as being particularly sensitive to tumor hypoxic microenvironment.

We conducted differential gene expression analysis on macrophage cluster 1 and combined COX and Lasso regression analysis to construct a novel hypoxia-related prognostic model, which contained 13 genes: *LYZ*, *SCN1B*, *PLAU*, *INSIG2*, *DSC2*, *MICAL1*, *U2AF1*, *KRTCAP2*, *DDX60L*, *SATB1*, *SAMD9*, *LTC4S* and *IGLL5*. Our hypoxia model shares common genes, such as *PLAU*, with other hypoxia-related prognostic models [27–29]. However, unlike other models constructed from bulk RNA data, our model was developed using single-cell data. This approach allowed us to identify the cell subpopulations with the highest hypoxia responsiveness in pancreatic cancer, providing a more precise localization of hypoxia-driven effects.

Based on our model, we calculated hypoxia score for each patient in the TCGA-PAAD cohort. Patients were then stratified into high and low hypoxia groups based on the median score. Our analysis revealed that patients in the high hypoxia group exhibited significantly worse prognostic outcomes compared to those in the low hypoxia group. Moreover, our hypoxia model demonstrated superior predictive performance for patient survival at 1, 2, and 3 years when compared to clinicopathologic characteristics. By integrating hypoxia scores with clinicopathologic features, our findings confirmed that the hypoxia score served as an independent prognostic factor. Interestingly, there were no significant differences in clinicopathologic features between the high and low hypoxia score groups. Moreover, within most subgroups defined by clinicopathologic characteristics, patients with high hypoxia scores generally exhibited worse prognoses compared to those with low scores. These findings underscored the predictive efficacy of the hypoxia model and was entirely independent of clinicopathologic features.

To further elucidate the relationship between the hypoxia microenvironment and tumor-infiltrating immune cells in pancreatic cancer, we employed several algorithms to assess immune infiltration states [30–32]. Our findings indicated a significant reduction in naïve B cells, which are well known for their anti-tumor immune responses, in patients with high hypoxia scores. This reduction potentially facilitated tumor immune evasion and progression, contributing to the substantially decreased survival rates observed in the high hypoxia group. Similar findings have been reported in studies of kidney renal clear cell carcinoma (KIRC), where cuproptosis signatures are closely associated with tumor immune cell infiltration and responses to immunotherapy, serving as a potential biomarker for predicting patient prognosis [33].

Pan-cancer analysis has emerged as a powerful approach to uncover biomarkers and therapeutic targets that play critical roles across multiple cancer types, providing insights into shared molecular mechanisms and prognostic factors [34–36]. In our study, pan-cancer analysis had further demonstrated that these 13 hypoxia-related genes, particularly the *KRTCAP2* gene, were not only significantly associated with the prognosis of pancreatic cancer but also played critical roles across various malignancies. Keratinocyte-associated protein 2 (*KRTCAP2*) is involved in N-glycosylation processes. Previous research has shown that elevated levels of KRTCAP2 protein are associated with reduced infiltration of CD8^+^ T cells and CD68^+^ macrophages in hepatocellular carcinoma tissues [37]. Additionally, *KRTCAP2* has been shown to regulate tumor cell proliferation, differentiation, and carcinogenesis in gastric cancer [38]. Elevated expression of *KRTCAP2* has also been associated with unfavorable prognosis in uveal melanoma, further underscoring its role as a prognostic marker [39]. Similar observations have been made with other pan-cancer biomarkers [40,41], where individual gene can demonstrate significant prognostic value and associations with immune cell infiltration across multiple cancer types. Moreover, when comparing the gene expression levels of *KRTCAP2* between tumor tissues and adjacent normal tissues, as well as across different cancer stages, it was found that *KRTCAP2* was consistently overexpressed in tumor tissues compared to adjacent normal tissues across a broad spectrum of cancers. Its expression also escalated with advancing tumor stages. Based on these findings, we propose that *KRTCAP2* serves as a potential tumor biomarker and represents a promising target for therapeutic intervention.

Despite the promising findings obtained, our study also has some limitations that need to be addressed. Firstly, additional investigations into the protein expression levels of hypoxia-related genes are necessary to complement our findings. Secondly, our study lacks supporting cellular and animal experiments to confirm the regulatory mechanisms in pancreatic cancer. Therefore, more comprehensive research is essential to thoroughly assess the potential and applicability of our hypoxia model in future studies.

In conclusion, we developed a thirteen-gene hypoxia-related prognostic model for pancreatic cancer by integrating single-cell RNA sequencing and bulk RNA sequencing data. This prognostic signature not only served as an independent factor for predicting patient outcomes, but also offered important insights into immune cell infiltration and chemotherapy response. Further, it holds promise for identifying potential new biomarkers and therapeutic targets in pancreatic cancer.

## Supporting information

S1 Fig Validation of the hypoxia-related prognostic model in PACA-CA cohort. (A) Relationship between survival status and hypoxia score in PACA-CA cohort. (B) Kaplan−Meier curves of patients in high and low hypoxia groups. (C) ROC curves of hypoxia model for predicting the risk of death at 1, 2, and 3 years. (D) Time-AUC curves evaluating the predictive capacity of the hypoxia model and clinicopathologic features. (E) Decision curve analysis evaluating the benefit rate of patients receiving clinical treatment based on the hypoxia model and clinicopathologic features. Data sources: Panels A-E use the PACA-CA cohort.
(TIF)

S2 Fig Validation of the hypoxia-related prognostic model in PACA-AU cohort. (A) Relationship between survival status and hypoxia score in PACA-AU cohort. (B) Kaplan−Meier curves of patients in high and low hypoxia groups. (C) ROC curves of hypoxia model for predicting the risk of death at 1, 2, and 3 years. (D) Time-AUC curves evaluating the predictive capacity of the hypoxia model and clinicopathologic features. (E) Decision curve analysis evaluating the benefit rate of patients receiving clinical treatment based on the hypoxia model and clinicopathologic features. Data sources: Panels A-E use the PACA-AU cohort.
(TIF)

S3 Fig Clinicopathologic features of hypoxia model. (A, B) Multivariate Cox regression analysis of hypoxia model and clinicopathologic characteristics in PACA-CA (A) and PACA-AU (B) cohort. (C-F) Relationship between hypoxia score with age (C), gender (D), TNM stage (E) and T stage (F) of patients in TCGA-PAAD cohort. Data sources: Panel A use the PACA-CA cohort. Panel B use the PACA-AU cohort. Panels C-F use the TCGA-PAAD cohort.
(TIF)

S4 Fig The prognostic value of hypoxia model in age (A, B), gender (C, D), T stage (E, F), N stage (G, H) and TNM stage (I, J), respectively. Data sources: Panels A-J use the TCGA-PAAD cohort.
(TIF)

S1 Table The coefficient of each gene in the hypoxia model. (XLSX)
