# Supplementary figures and images for: Development of a hypoxia-responsive macrophage prognostic model using single-cell and bulk RNA sequencing in pancreatic cancer
Source: PLoS One. 2025 May 2;20(5):e0322618. doi: 10.1371/journal.pone.0322618 (PMC12047781; doi:10.1371/journal.pone.0322618)

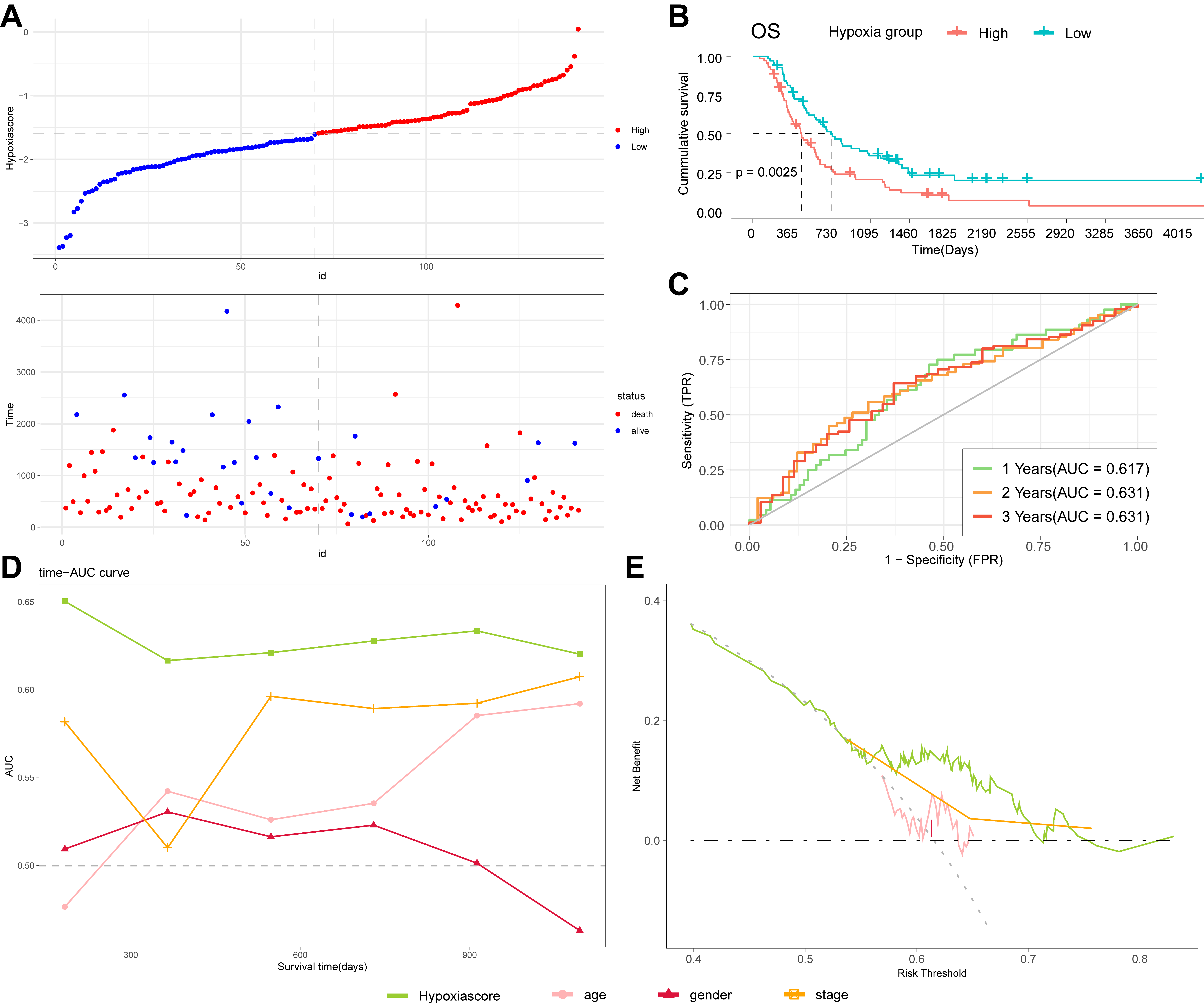

Supplement: S1 Fig — (A) Relationship between survival status and hypoxia score in PACA-CA cohort. (B) Kaplan−Meier curves of patients in high and low hypoxia groups. (C) ROC curves of hypoxia model for predicting the risk of death at 1, 2, and 3 years. (D) Time-AUC curves evaluating the predictive capacity of the hypoxia model and clinicopathologic features. (E) Decision curve analysis evaluating the benefit rate of patients receiving clinical treatment based on the hypoxia model and clinicopathologic features. Data sources: Panels A-E use the PACA-CA cohort. (TIF) [file pone.0322618.s001.tif]

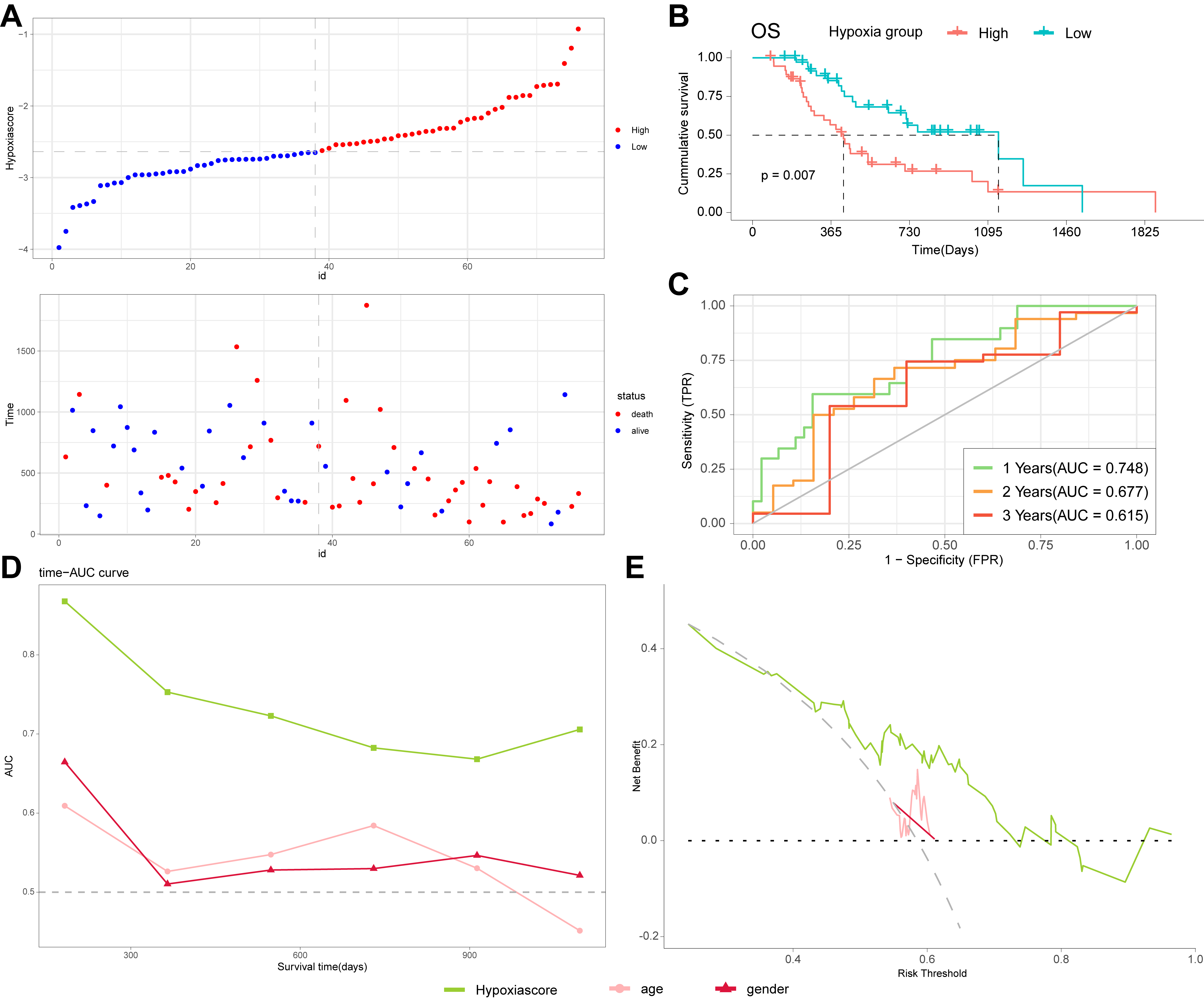

Supplement: S2 Fig — (A) Relationship between survival status and hypoxia score in PACA-AU cohort. (B) Kaplan−Meier curves of patients in high and low hypoxia groups. (C) ROC curves of hypoxia model for predicting the risk of death at 1, 2, and 3 years. (D) Time-AUC curves evaluating the predictive capacity of the hypoxia model and clinicopathologic features. (E) Decision curve analysis evaluating the benefit rate of patients receiving clinical treatment based on the hypoxia model and clinicopathologic features. Data sources: Panels A-E use the PACA-AU cohort. (TIF) [file pone.0322618.s002.tif]

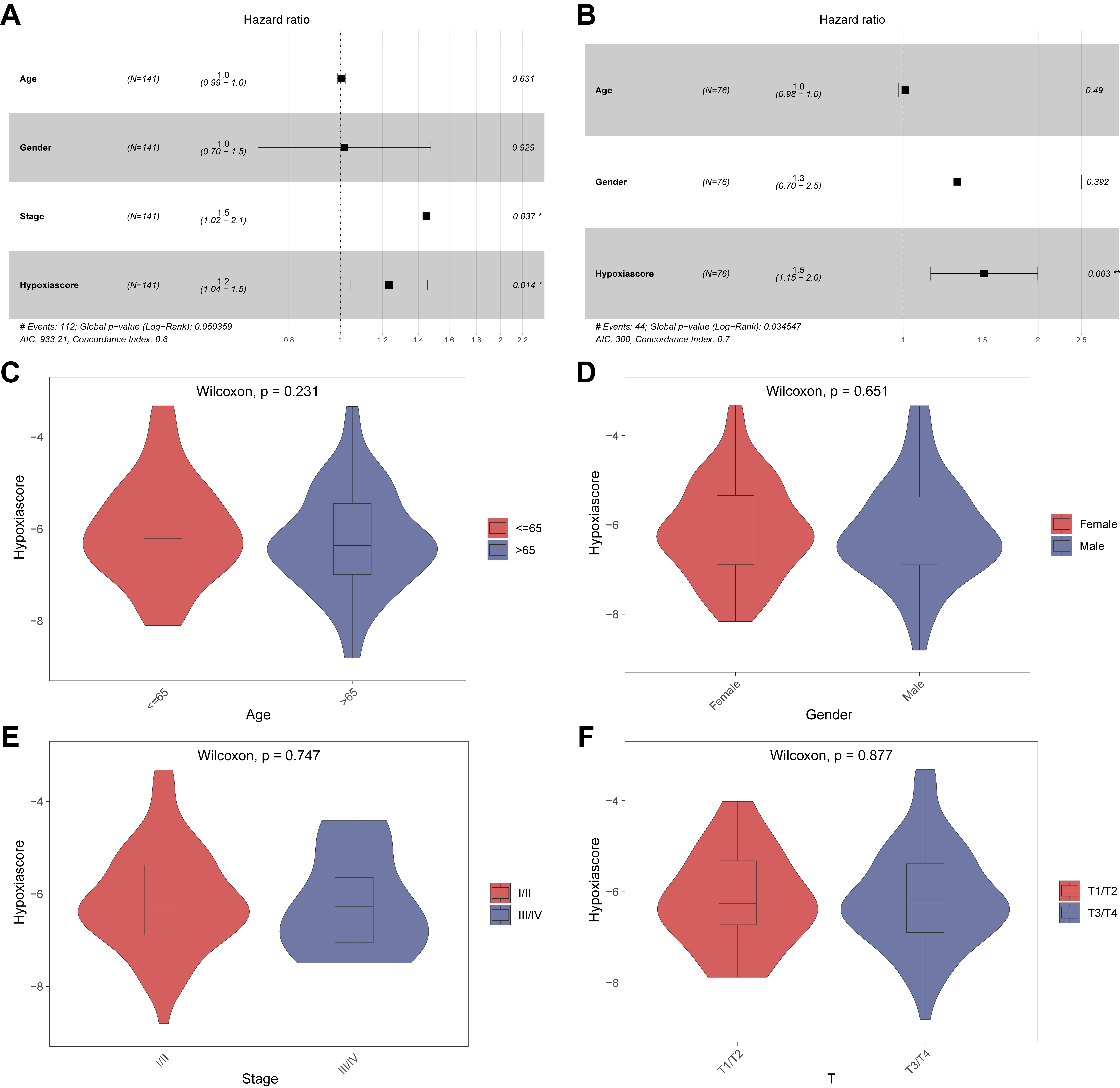

Supplement: S3 Fig — (A, B) Multivariate Cox regression analysis of hypoxia model and clinicopathologic characteristics in PACA-CA (A) and PACA-AU (B) cohort. (C-F) Relationship between hypoxia score with age (C), gender (D), TNM stage (E) and T stage (F) of patients in TCGA-PAAD cohort. Data sources: Panel A use the PACA-CA cohort. Panel B use the PACA-AU cohort. Panels C-F use the TCGA-PAAD cohort. (TIF) [file pone.0322618.s003.tif]

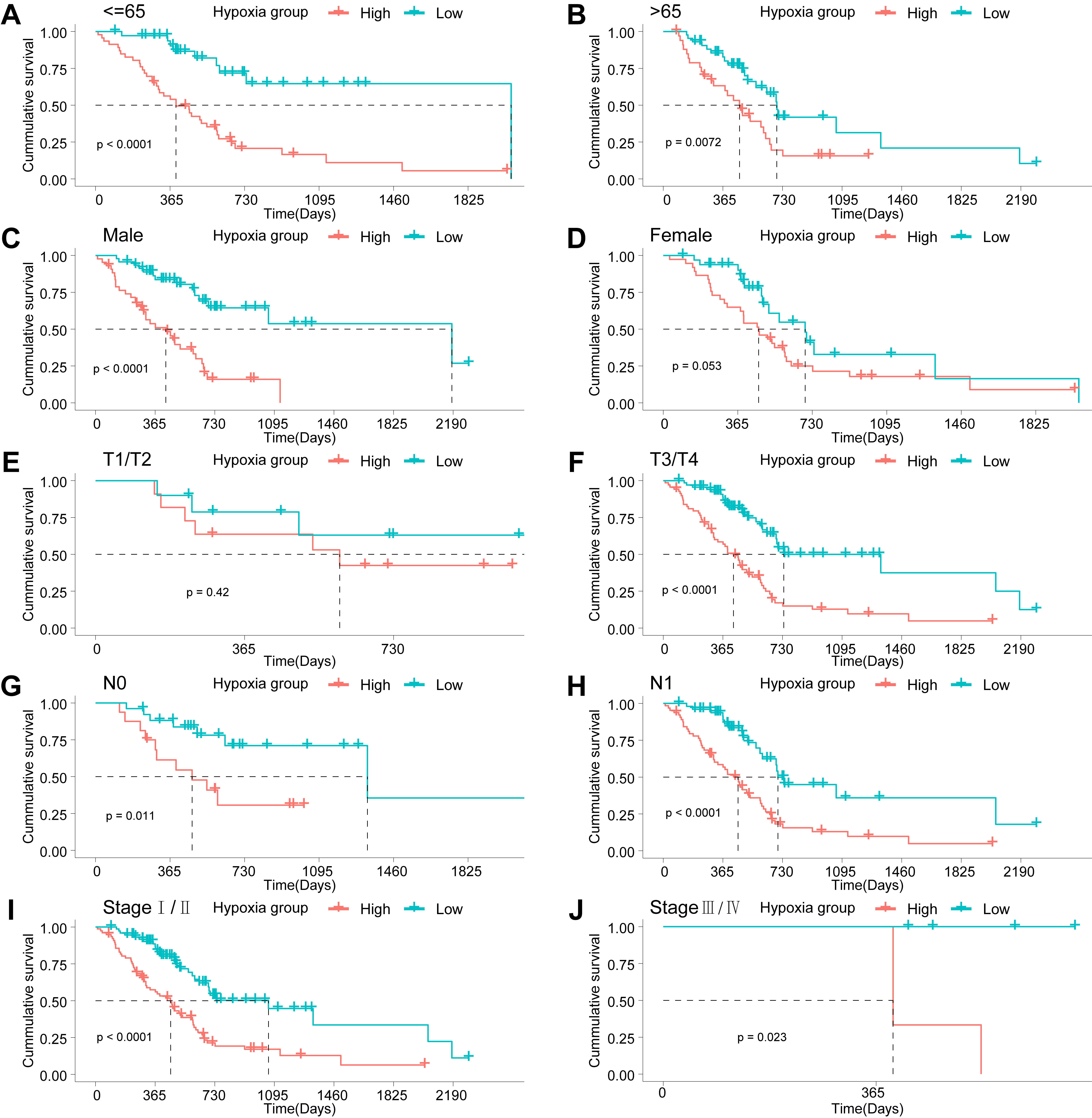

Supplement: S4 Fig — Data sources: Panels A-J use the TCGA-PAAD cohort. (TIF) [file pone.0322618.s004.tif]
